# Chromatin Interaction Analysis with Paired-End Tag (ChIA-PET) sequencing technology and application

**DOI:** 10.1186/1471-2164-15-S12-S11

**Published:** 2014-12-19

**Authors:** Guoliang Li, Liuyang Cai, Huidan Chang, Ping Hong, Qiangwei Zhou, Ekaterina V Kulakova, Nikolay A Kolchanov, Yijun Ruan

**Affiliations:** 1 National Key Laboratory of Crop Genetic Improvement, College of Informatics, Huazhong Agricultural University, 1, Shizishan Street, Wuhan, 430070, China; 2 Institute of Cytology and Genetics SB RAS, Lavrentyeva, 10, Novosibirsk, 630090, Russia; 3 Novosibirsk State University, Pirogova, 2, Novosibirsk, 630090, Russia; 4 The Jackson Laboratory for Genomic Medicine, 10 Discovery Drive, Farmington, CT 06032, USA

## Abstract

**Background:**

Long-range chromatin interactions play an important role in transcription regulation. Chromatin Interaction Analysis with Paired-End-Tag sequencing (ChIA-PET) is an emerging technology that has unique advantages in chromatin interaction analysis, and thus provides insight into the study of transcription regulation.

**Results:**

This article introduces the experimental protocol and data analysis process of ChIA-PET, as well as discusses some applications using this technology. It also unveils the direction of future studies based on this technology.

**Conclusions:**

Overall we show that ChIA-PET is the cornerstone to explore the three-dimensional (3D) chromatin structure, and certainly will lead the forthcoming wave of 3D genomics studies.

## Background

**Figure 1:**
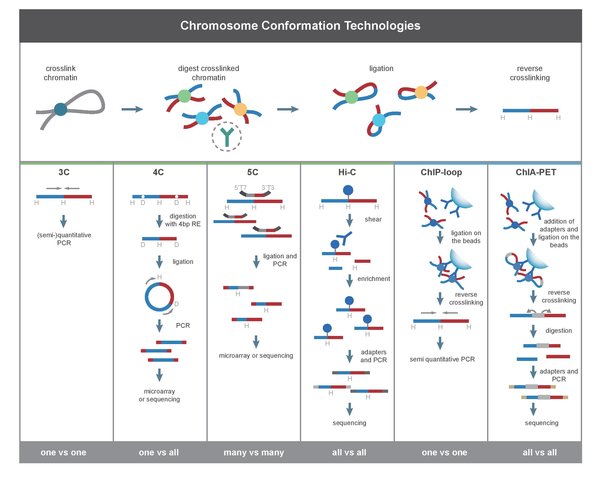
**Comparison among 3C and its derived methods**. This figure is from De Wit and de Laat, 2012 [8].

Transcription regulation is a complex yet well-organized process in eukaryotes, in which chromatin interactions play a critical role and thus serve to regulate gene expression as well as further influence other cellular activities. Many technologies have been developed to study the binding of transcription factors (TF) for transcription regulation, such as chromatin immunoprecipitation (ChIP) microarray (ChIP-chip) [1], ChIP-PET [2] and ChIP-Seq [3], but they are unable to determine the target genes of the distal TF binding sites. Another challenge is to define whether such distal binding sites are functional, i.e. physically proximal to target gene promoters via chromosome loops or attracting RNA polymerase II complex for gene transcription. Therefore, identification of genome-wide distal chromatin interactions that lead the regulatory elements to their target genes may provide novel insights into the study of transcription regulation. Chromosome conformation capture (3C) [4] and its derivatives, 4C [5,6] and 5C [7] can reveal long-range chromatin interactions involved in transcription regulation, but these techniques are limited either because they are low-throughout, such as 3C, or they can't map interacting regions with high resolution in the whole genome [8] (Figure 1). In this case, it is desired to have a method capable of analyzing chromatin interactions on genome level with high throughput and high resolution.

Chromatin Interaction Analysis with Paired-End-Tag sequencing (ChIA-PET) method [9] fits these demands. It is an unbiased, genome-wide, high-throughput and *de novo *method. Compared with Hi-C [10], another emerging method for chromatin interactions at a global scale, ChIA-PET is better at its higher resolution associated with a protein of interest for functional study, and lays a solid foundation for studying long-range chromatin interactions in a three-dimensional (3D) manner, as well as provides a more reliable way to determine TF binding sites and identify chromatin interactions.

Till now, ChIA-PET has been successfully applied to human MCF7 cells [9], human cancer cells [11], human T cells [12], mouse embryonic stem cells [13-15], mouse neural progenitor cells [15], and mouse B cells [16], and other cells [17-19], referring to Additional file 1 Table 1 for the available list of ChIA-PET applications by our best knowledge. Li et al. [11] has identified three kinds of interactions, named enhancer-promoter, enhancer-enhancer and promoter-promoter interactions, and demonstrated that over 40% of enhancers don't regulate their nearest promoters. Sandhu et al. [20] has proposed the concept of "chromatin interaction networks", showing the phenomenon that human genome converges to a scale-free and hierarchical network, through which functions are enriched in the so-called "chromatin communities". This work unveiled the chromatin interactions in high order architectures; these architectures may act as transition from linear map to 3D/4D genome studies. Several protein associated interactions like ER-α, RNA polymerase II (RNAPII), CTCF and SMCIA have been studied to investigate how remote regulators interact with their target promoters.

In this paper, we will introduce the experimental protocol and data analysis procedure of ChIA-PET technology in comparison with other genome methods for chromosome conformation studies, and discuss the applications of ChIA-PET technology on different proteins and human/ mouse cells.

## Experimental process

**Figure 2:**
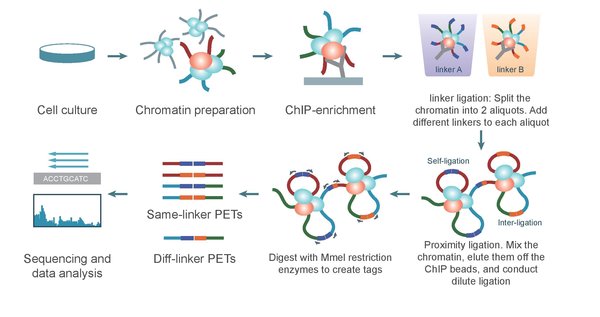
**The ChIA-PET experimental protocol, which includes chromatin preparation, ChIP, linker ligation, proximity ligation, *Mme*I restriction digestion, and DNA sequencing**. This figure is from Li et al., 2010 [22].

**Figure 3:**
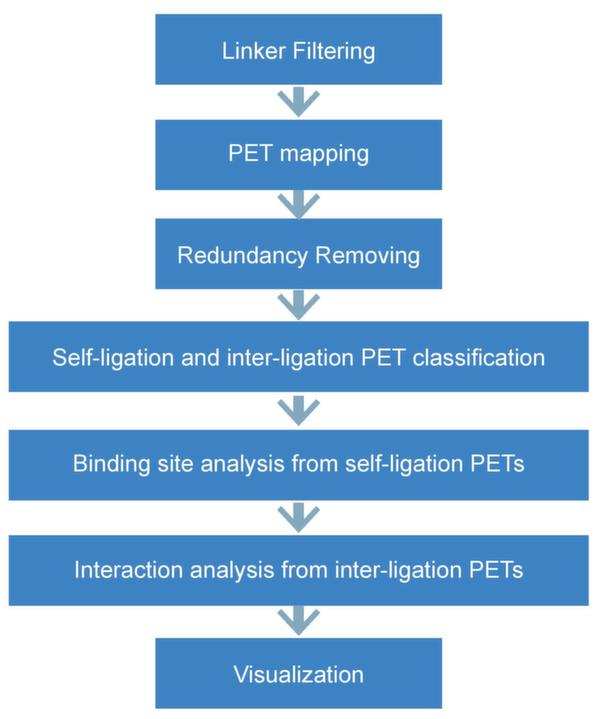
**Fundamental steps to analyze ChIA-PET data**.

Compared with other 3C-derived technologies, ChIA-PET protocol is a complex process. It can be summarized into three parts: wet-lab experiments (Figure 2), data analysis (dry-lab experiments, Figure 3) and experimental verification.

First, the ChIA-PET wet lab complies with the ChIP experiment [21]. Like ChIP-Seq experiment, formaldehyde is used to crosslink DNA-protein complexes in the nucleus and followed by breaking the complexes into fragments with sonication. Then, ChIP is used to enrich DNA fragments bound by a protein of interest. Next, DNA fragments in ChIP-enriched chromatin complexes are ligated with two different half-linker oligonucleotides in two aliquots. Then, the two aliquots are mixed and proximal half-linkers would be ligated with each other. After reverse crosslinking, the proteins in the complexes are digested and the DNA fragments are extracted. After digestion with restriction enzyme *Mme*I, DNA fragments form paired-end tags (PETs) constructs, in "tag-linker-tag" order. Eventually, the PETs are taken to sequencing with new-generation sequencing facilities, like Illumina Hi-Seq2500. The sequence reads are aligned to the reference genome and further analyses are performed to reveal long-range interactions between functional elements. During the whole experiments, the quality of DNA that is enriched by ChIP is the critical part. And the essence of ChIA-PET is the linker's design. The linkers not only have *Mme*I restriction site that could cut 20 bp outward, but also have barcodes to estimate the noise caused by random ligation.

The results of pair-end sequencing are stored in two fastq files, which can be processed with ChIA-PET Tool [22] or other methods [23]. Generally, there are seven steps in ChIA-PET data processing (Figure 3): 1) linker filtering, 2) PET mapping, 3) redundancy removing, 4) self-ligation and inter-ligation PET classification, 5) binding site analysis with self-ligation PETs, 6) chromatin interaction analysis with inter-ligation PETs, and 7) visualization of the chromatin interaction data. In the first step, linkers will be aligned to the reference half-linker nucleotide sequences. There are two kinds of half-linkers, named as A and B, which have the same nucleotides, except the barcodes. Therefore, PETs are classified into two categories based on the linker compositions: same linkers (AA or BB) and different linkers (AB or BA). Then linkers are excluded from the raw reads and the remaining DNA fragments are kept for further analysis. After linker filtering, the short DNA sequences are aligned to reference genome using BWA [24], Bowtie [25], BatMis [26], or other mapping tools. With SAMtools [27] and BEDtools [28], the redundant and low quality mapping sequences are filtered out. Next, PETs can be divided into self-ligation PETs and inter-ligation PETs. Self-ligation PETs refer to the reads from individual DNA fragments looping through their both ends and are mapped to genome within a short distance on the same chromosome. Inter-ligation PETs refer to the reads from different DNA fragments, and are generally with both tags mapped in different chromosomes or in long distance in the same chromosome. While self-ligation PETs are used to figure out protein binding sites on the genome, inter-ligation PETs can predict the chromatin interactions by clustering. Then we also have to make sure the interaction clusters between two anchors really exist or just occur by chance. Li et al. [22] used Fisher's exact test based on hypergeometric distribution to quantify the interaction frequency. Recently, Paulsen et al. [23] proposed a new statistical model based upon non-central hypergeometric distribution, which takes genome distance-dependent relationships into account for p-value estimation. Finally, ChIA-PET browser can be built to report data and visualize binding sites as well as interaction clusters.

The interactions obtained by data processing require validation with wet-lab experiment. The interactions between DNA elements in short genomic distance can be validated through 3C experiment. As for DNA fragments in long-range interactions (two anchors located in different chromosomes or in the same chromosome with more than 1 million base pair away), we could use microscope technique like DNA Fluorescence in situ hybridization (DNA-FISH) [29] to directly observe the location of interaction anchors and relative spatial distance in the nucleus.

## Applications of ChIA-PET technology

### Interactions between DNA elements mediated by TFs

**Figure 4:**
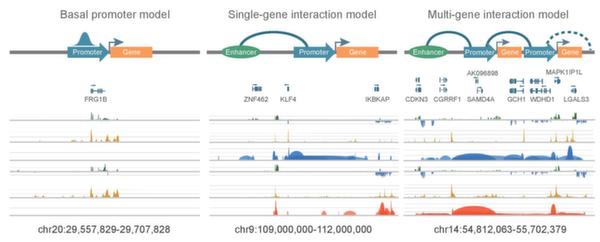
**Transcription models based on chromatin interactions**. Left to right: basal promoter model, single-gene interaction model and multi-gene interaction model. This figure is from Li et al., 2012 [11]

While ChIP-Seq is used to analyze the interactions between DNA and protein, ChIA-PET works on the interactions between DNA fragments fundamentally. Fullwood et al. [9] used ChIA-PET technology to construct chromatin interaction network bound by estrogen receptor α (ER-α) from human breast cancer cell line MCF7 and found long-range ER-α binding sites are mostly located at promoter regions. Handoko et al. [13] found the CTCF-mediated interactions from mouse embryonic pluripotent stem cells. Five distinct chromatin domains revealed by CTCF ChIA-PET raised a new model of CTCF function for chromosome structure organization and linking enhancers to promoters for gene transcription regulation. Li et al. [11] detected promoter-centered distant interactions bound by RNAPII in cancer cells. In addition to promoter-enhancer and enhancer-enhancer interactions, they found that promoter-promoter interactions are also pervasive in human cells. In all the promoter-nonpromoter interactions, more than 40% of the non-promoter regulatory elements didn't interact with their nearest promoters. This means that the current assumption in ChIP-Seq study - the transcription factor binding sites regulate their nearest genes - is not valid. Based on the RNAPII-mediated chromatin interactions, especially promoter-promoter interactions, they proposed three types of transcription models (Figure 4): 1) basal promoter models, in which the gene promoter regions are enriched with RNAPII, but are not involved with any chromatin interactions; 2) single-gene interaction models, in which one gene is involved with one or more promoter-nonpromoter interactions; and 3) multi-gene interaction models, in which multiple genes are linked together by chromatin interactions to form a transcription factory for potential correlated transcription. Zhang et al. [15] figured out the interactions mediated by RNAPII in mouse pluripotent embryonic stem cells, neural stem cells and neural progenitor cells. 40,000 long-range interactions from their work uncovered the associations between promoters and distal-acting enhancers. Demare et al. [18] first described cohesin-associated chromatin interactions on the genome-wide study by ChIA-PET. 65% of interactions bound by cohesin subunit SMC1A are co-occupied by CTCF. Analyzing the interactions in multiple tissues, they proposed a potential model of tissue-specific gene regulation.

After characterizing enhancer-promoter interactions in human T cells, Chepelev et al. [12] presented that enhancers increase the expression of their target genes in a cell-specific way, and interacting promoters are co-expressed. In addition, chromosomes in nucleus are organized in multiple levels to perform functions, and many factors besides CTCF may be involved in this process in T cells. The detailed mechanism needs to be addressed in future studies.

He et al. [30] created an efficient "classifier", calculating the possibility of DNA looping based on ER-α binding peaks obtained by ChIP-Seq, and then predicted chromatin interactions mediated by ER-α. This is the first work using ChIP-Seq to predict chromatin interactions, providing a supplement to ChIA-PET.

### Chromatin interaction network

Cell is a complex system, and its functional characteristics rely on the cross talks of constituents including DNA, RNA, proteins, etc. Biological scientists have tried to use network approaches to build frameworks of these interactions. Many biological networks, such as metabolic networks, protein-protein interaction networks have been widely studied. However, networks of chromatin interactions are not known till recently because the lack of proper genome-wide datasets. Now data generated by ChIA-PET is unfolding the study of chromatin interaction networks.

**Figure 5:**
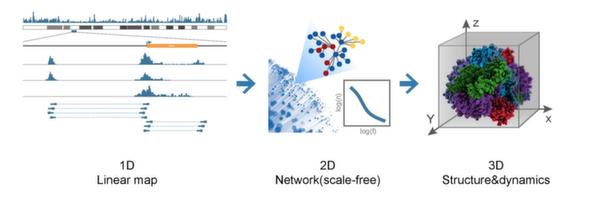
**Transformation from current chromatin interactions to future 3D/4D dynamics**. This figure is from Sandhu et al., 2011 [31].

Anchors in different interacting pairs are not isolated - they may overlap with or link to other counterparts in different physical regions. This leads to the concept of "chromatin interaction networks" [31], which refers to the 2D structure of genome-wide chromatin interaction (Figure 5). Like many cellular networks, chromatin interaction network [20] has scale-free and modular topology - most nodes participate in only one or two interactions, while a few nodes, known as "hubs", connect with a disproportionately high number of nodes. These "hubs" prefer to link to each other and conform "rich-club", which carry out essential cellular functions and contribute to the robustness of the network. Chromatin interaction network is organized into "community", and genes within community perform related functions and respond to external stimuli in a coordinated manner, which means these communities may have been shaped during millions of years' evolution.

This approach has also been applied to interactions between microRNA genes. MicroRNAs are small noncoding RNAs that play a key role in transcription regulation. Chen et al. [32] has built chromatin interaction networks involving both microRNAs and protein-coding genes. The network consists of 2292 communities, and these communities are enriched in fundamental cellular functions. The disease-related microRNAs tend to interact with each other.

In future studies, we can not only apply the method to other specific kinds of genes, but also combine interdependent networks, since cellular activities happen together and link to each other. Also, chromatin interaction networks may lay the foundation of 3D or even 4D genome waves, which transfer from static to dynamic [31].

### Functional studies of chromatin interactions

There are several ways to study the functions of chromatin interactions identified by ChIA-PET: 1) luciferase reporter gene assay [11]; 2) knock-down experiment for the expression level of the protein of interest [11]; 3) enhancer assay from transgenic experiments for the identified regulatory elements [15]; and 4) genomic editing methods (such as zinc-finger nuclease genome editing, TALENs and CRISPR/Cas9) to perturb the chromatin interactions [16]. We will take the luciferase reporter gene assay and the TALENs as examples.

**Figure 6:**
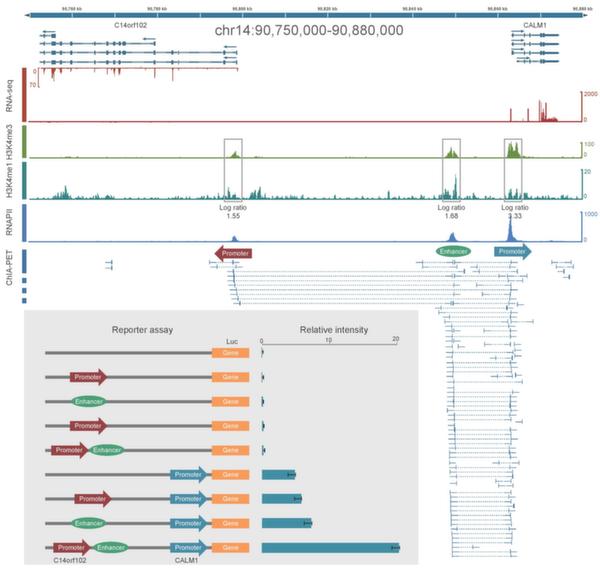
**Luciferase reporter gene assay experiments for interactions at C14orf102-CALM1 locus including both promoters and enhancer**. This figure is adopted from Li et al., 2012 [11].

In Figure 6, ChIA-PET data shows that there are chromatin interactions between promoter regions of gene C14orf102 and CALM1, and also there are interactions between a distal regulatory element and CALM1 promoter. The distal regulatory element is categorized as an enhancer because it is enriched with histone mark H3K4me1. This means that the CALM1 promoter interacts with two other regions: C14orf102 promoter region and the distal enhancer. The luciferase reporter gene assay with interaction anchors shows that 1) without promoter, the luciferase reporter assay only has marginal expression level, with/without other regulatory elements; 2) with CALM1 promoter sequence alone, the luciferase reporter assay has basal expression level; 3) with promoter and one of the regulatory elements, the expression level in the luciferase reporter assay only increases marginally; and 4) with both regulatory elements, the expression level in the luciferase assay increases more than 3 folds compared with the expression level from assay with promoter alone. This means that there are combinatorial effects in the regulatory elements for gene expression regulation [11].

**Figure 7:**
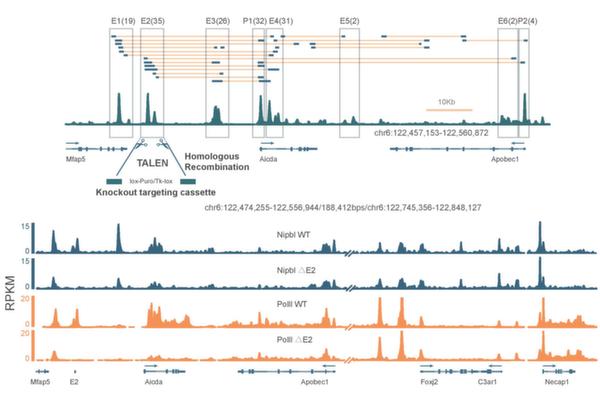
**TALEN experiments for interactions around gene Aicda**. This figure is adopted from Kieffer-Kwon et al., 2013 [16].

In Figure 7, the upper panel shows that there are six enhancer regions linked to the promoter region of gene Aicda in mouse B cells, and the enhancer E2 was selected as the region to knock out by TALEN experiment. The lower panel shows that the RNAPII and Nipbl binding intensities at the Aicda promoter region are much reduced in the genetically modified cells, compared to the binding intensities in the wild-type cells. This means that the enhancer E2 really influence the transcription regulation of gene Aicda.

### Reconstruction of the 3D structure of chromatin

A precise three-dimensional structure of chromatin provides a better landscape of the biological functions. So far, the data of remote interaction is suitable to reconstruct the 3D genome structure. Two 3C derivations, namely Hi-C [10] and ChIA-PET [9], actually reflect the structure of the whole genome. The Hi-C technology could capture all the interactions but with low resolution. The ChIA-PET technology greatly enhances the resolution but it can only identify the interactions mediated by a known protein. So, ChIA-PET data can be used to conduct more intense modeling.

Much progress has been made in building 3D chromosomal structure from Hi-C data [33-35]. However, there is no published article to build 3D genome structure from ChIA-PET data yet. But some research institutions try to work on it. The methods to reconstruct the 3D structure with chromatin interaction data are based on the assumption that, if the interaction frequency between two loci is high, the spatial distance between the two loci is proximal and vice versa. There are two major approaches for modeling the 3D structure of chromatin [36]: one is physical model such as bead-on-a-string model to explain the results of the experiment, and another one is the nonlinear optimization model to reconstruct the structure. Many physical properties must be taken into consideration in the first method. The first step of the nonlinear optimization model to reconstruct the structure is to convert the chromatin interaction frequency into spatial distance and then convert the spatial distance into three-dimensional structure. Of course we should do the normalization before applying the sequencing data for chromatin 3D structure reconstruction, because of the various biases in the experiment and the data, such as GC bias, mappability [37]. For a lack of direct parameters to evaluate the established 3D structure in a genome-wide scale, the development of electron microscopy will have a great role in the promotion of the research on 3D structure of chromatin. The visualization of chromatin interactions combined with functional assays is an important way to give people a more intuitive impression of the genome structure and a comprehensive understanding of the function of the genome.

## Discussion and perspectives

ChIA-PET has been successfully applied for transcription regulation analysis in a number of studies and different chromatin interaction models have been identified. Still, there are spaces to improve the ChIA-PET protocol and analysis pipeline - to make the protocol more concise and easy to conduct, and the data analysis process more automatic and customizable.

As for the method development, the followings are several aspects to be considered.

1) Reduce the number of cells required for ChIA-PET experiments. Currently, tens of millions of cells are required in the ChIA-PET experiments. This is fine for many man-made cell lines, which could be cultured in the laboratory for more cells. However, in many real applications, like tissues from patients, there are only limited numbers of cells. To make ChIA-PET applicable to such real situations, we need to reduce the number of cells required in the experiment.

2) Make the DNA fragments longer. In the current ChIA-PET protocol, restriction enzyme MmeI is used to digest the ligated DNAs to prepare the DNA constructs, and only 20 nucleotides from the DNA fragments are kept for further analysis, which is quite short, especially for the genome with high repetitive sequence. We need to make the DNA fragment longer for more specific mapping of the reads to the reference genome.

3) Improve the comprehensiveness of the current pipeline for ChIA-PET data processing. Currently, the ChIA-PET Tool can perform the fundamental process of the ChIA-PET data. Methods for more comprehensive analysis of ChIA-PET data are not publically available. This hinders the application of ChIA-PET.

4) 3D genome structure reconstruction. At present, interaction data generated by ChIA-PET and Hi-C are mainly exhibited through 2D graphs, which is far away from 3D visualization. We need to reconstruct the 3D structure of the genome inside the nucleus to demonstrate the chromatin interactions in the real spatial manner[38].

5) Large-scale validation of chromatin interactions and verification of their functions. Resolution of DNA-FISH needs to be enhanced to get more precise result. Observation of exquisite structure of the whole genome is still a long-term task, considering cell is in a dynamic state. Tens of thousands of chromatin interactions have already been reported by ChIA-PET, but only tens to hundreds have been validated with DNA-FISH and much more needs to be done. In addition, the functions of such interactions need to be examined one by one, with different technologies. The recent progress in genome editing technology CRISPR/Cas9 makes it a promising choice for such a task.

6) The field of transcription regulation focuses on the exploration of relationships between DNA, RNA and protein separately. We need new methods to demonstrate how these factors work together to regulate gene transcription, not just with partial information.

In addition to ChIA-PET method development, Hi-C method and applications have shown some interesting progress. Gavrilov et al. [39] discussed variability of chromosome contacts between individual cells and suggested in-gel selection of contacting genome fragments. Thévenin et al. [40] show using Hi-C contact map that chromosomal segments close in the 3D space of the nucleus tend to contain genes - members of the same functional group. It is expected that the combination of low-resolution structure from Hi-C and high-resolution structure from ChIA-PET will inspire more insights about chromatin structures and their functions in biology.

In the near future, we hope the development of the new technologies will fulfill all the mentioned considerations and lead to novel insights about the mechanism for gene transcription regulation.

## Abbreviations

ChIP-PET: Chromatin ImmunoPrecipitation - Paired-End Tag; ChIA-Seq: Chromatin ImmunoPrecipitation with sequencing; ChIA-PET: Chromatin Interaction Analysis with Paired-End Tag sequencing; RNAPII: RNA Polymerase II.

## Funding

GL was supported by the Fundamental Research Funds for the Central Universities [2662014PY001]. KEV and NAK were supported by the RSF grant 14-24-00123.

## Competing interests

The authors declare that they have no competing interests.

## Authors' contributions

GL conceived and coordinated the project. GL, LC, HC, PH, and QZ wrote this manuscript, with contribution from others.

## Supplementary Material

Additional file 1 **List of published ChIA-PET data**. Click here for file

## Declarations

Publication of this article has been funded by the Russian Science Foundation (RSF 14-24-00123).

This article has been published as part of *BMC Genomics *Volume 15 Supplement 12, 2014: Selected articles from the IX International Conference on the Bioinformatics of Genome Regulation and Structure\Systems Biology (BGRS\SB-2014): Genomics. The full contents of the supplement are available online at http://www.biomedcentral.com/bmcgenomics/supplements/15/S12.
